# Retraction Note: Ras-ERK1/2 signaling contributes to the development of colorectal cancer via regulating H3K9ac

**DOI:** 10.1186/s12885-021-08340-3

**Published:** 2021-05-24

**Authors:** Peng Tian, Yanfei Zhu, Chao Zhang, Xinyu Guo, Peng Zhang, Huanzhou Xue

**Affiliations:** 1 Department of Gastrointestinal Surgery, Zhengzhou University People’s Hospital (Henan Provincial People’s Hospital), Zhengzhou, 450003 China; 2 Department of General Surgery, Wuxi People’s Hospital of Nanjing Medical University, Wuxi, 214023 China; 3 Department of General Surgery, Zhengzhou University People’s Hospital (Henan Provincial People’s Hospital), No.7, Weiwu Road, Zhengzhou, 450003 Henan China

**Retraction Note: BMC Cancer 18, 1286 (2018)**

**https://doi.org/10.1186/s12885-018-5199-3**

The Editor in Chief retracted this article because of significant concerns regarding a number of Figures presented in this work, which question the integrity of the data. Specifically, a number of images appear to be the same and rotated between Fig. 2C, 2D and 4D in this article, and Fig. 2 and 3 in an article published earlier by unrelated authors ([1], now retracted). In addition, it appears that one image was re-used between Fig. 2D and 4D within this article.

The Editor-in-Chief therefore no longer has confidence in the integrity of the data in this article.

The authors have not responded to any correspondence from the editor or publisher about this retraction.
